# The Effectiveness of Intermittent Theta Burst Stimulation for Stroke Patients With Upper Limb Impairments: A Systematic Review and Meta-Analysis

**DOI:** 10.3389/fneur.2022.896651

**Published:** 2022-07-06

**Authors:** Wenhao Huang, Jiayi Chen, Yadan Zheng, Jin Zhang, Xin Li, Liujie Su, Yinying Li, Zulin Dou

**Affiliations:** ^1^Department of Rehabilitation Medicine, The Third Affiliated Hospital of Sun Yat-sen University, Guangzhou, China; ^2^Guangzhou University of Chinese Medicine, Guangzhou, China

**Keywords:** iTBS, stroke, upper limb function, systematic review, meta-analysis

## Abstract

**Background:**

Upper limb impairments are one of the most common health problems of stroke, affecting both motor function and independence in daily life. It has been demonstrated that intermittent theta burst stimulation (iTBS) increases brain excitability and improves upper limb function. Our study sought to determine the role of iTBS in stroke recovery.

**Objective:**

The purpose of this study was to determine the efficacy of iTBS in individuals with upper limb impairments following stroke.

**Methods:**

The databases used included Cumulative Index to PubMed, EMBASE, ESCBOhost, The Cochrane Library, Chinese Biomedical Database, Web of Science, China Biology Medicine (CBM), China National Knowledge Infrastructure (CNKI), Technology Periodical Database (VIP), and WanFang Database. Studies published before November 2021 were included. Each participant received an iTBS-based intervention aimed at improving activity levels or impairment, which was compared to usual care, a sham intervention, or another intervention. The primary outcome measure was a change in upper limb function assessment. Secondary outcomes included impairment, participation, and quality of life measures.

**Result:**

A total of 18 studies (*n* = 401 participants) that met the inclusion criteria were included in this study. There was a slight change in the upper limb function of the iTBS group compared with the control group, as measured by the Fugl-Meyer Assessment-Upper Extremity (FMA-UE) score (mean difference 2.70, 95% CI −0.02 to 5.42, *p* = 0.05). Significant improvement in resting motor threshold (RMT) and motor-evoked potential (MEP) was also observed in the meta-analysis of iTBS (MD 3.46, 95% CI 2.63 to 4.28, *p* < 0.00001); (MD 1.34, 95% CI 1.17 to 1.51, *P* < 0.00001). In addition, we got similar results when the studies were using the Modified Barthel Index (MBI) assessment (mean difference of 7.34, 95% CI 0.47 to 14.21, *p* = 0.04).

**Conclusion:**

Our study established the efficacy of iTBS in improving motor cortical plasticity, motor function, and daily functioning in stroke patients. However, the review requires evidence from additional randomized controlled trials and high-quality research.

**Systematic Review Registration:**

https://www.crd.york.ac.uk/PROSPERO/

## Introduction

Stroke is one of the leading causes of long-term disability in adults and one of the leading causes of death (1). Survivors of strokes often suffer from a variety of sequelae that impact their daily lives. Upper extremity dysfunction is one of the most common post-stroke problems and is a serious impairment in which patients are unable to perform normal activities of daily living because they cannot control their hands dexterously. Therefore, restoring upper extremity dysfunction is particularly critical to stroke treatment.

Repetitive transcranial magnetic stimulation (rTMS) is a non-invasive method for stimulating specific brain areas that have been shown to be useful in stroke rehabilitation (2). Nowadays, rTMS has been widely used to treat motor, speech, and cognitive dysfunctions (3). Theta burst stimulation (TBS) is a new type of rTMS that can elicit cortical plastic changes by varying the stimulation intensity. TBS's advantages include its short duration and low-intensity stimulus pulses, which make it more acceptable to participants than some other non-invasive brain-stimulating protocols (4). TBS is classified into two major subtypes: intermittent theta burst stimulation (iTBS) and continuous theta burst stimulation (cTBS) (5). By stimulating the cerebral cortex with TBS, iTBS can produce a long-term potentiation-like effect while maintaining an excitatory effect on the cortex. In comparison, cTBS can cause long-lasting depression and develop an inhibitory effect (6). In particular, iTBS may increase excitability by reducing perisomatic inhibition of pyramidal cells by parvalbumin+ (PV+) fast-spiking interneurons (4). Numerous studies have revealed that iTBS is now more frequently used and that its function has improved significantly as a result of its use. According to Platz and Thomas, priming M1 or S1 with excitatory rTMS as iTBS improved motor learning across a range of sensorimotor abilities (7). On the contrary, a study found that cTBS applied to the contralesional sensorimotor cortex had no additional effect on the primary outcome measures of motor function or skilled motor task performance (8). Another study found that participants who received cTBS had decreased exercise-related motor performance and retention during shorter exercise practice trials (9). As a result, this study focused on the effect of iTBS on upper-limb functional recovery instead of cTBS.

Recent studies have been conducted to determine the effects of TBS on upper limb motor dysfunction following stroke, but the results are controversial (6). According to an iTBS article, it could alter the excitability of the motor cortex (10). The effect on upper limb function in stroke patients, on the contrary, has been uncertain. Meanwhile, the majority of meta-analyses have concentrated exclusively on the effect of rTMS on upper limb function, ignoring the role of iTBS. A comprehensive review and meta-analysis were conducted to demonstrate that rTMS and iTBS could be used to treat post-stroke spasticity using the modified Ashworth index (11). Besides, although iTBS has been used clinically, it is not used commonly, and its effectiveness has yet to be confirmed (12). As a result, we conducted a systematic review and meta-analysis to identify data supporting the use of iTBS to facilitate stroke patients in regaining control of their injured hands. Our systematic review of the literature would identify the PICO question, “Can iTBS help stroke patients in regaining upper limb function?”. The answers may enhance new thinking in occupational therapy and help determine the effectiveness of iTBS.

## Method

### Protocol and Registration

This review followed the Preferred Reporting Items for Systematic Reviews and Meta-Analyses (PRISMA) guidelines, and the protocol was registered in the PROSPERO database (registration number: CRD42021282832) (13).

### Search Strategy

A complete literature search was conducted using PubMed, EMBASE, ESCBOhost, The Cochrane Library, Chinese Biomedical Database, Web of Science, China Biology Medicine (CBM), China National Knowledge Infrastructure (CNKI), Technology Periodical Database (VIP), and WanFang Database. The searching key terms included “Cerebrovascular Disorders” OR “Brain Ischemia” OR “Cerebral Hemorrhage” OR “stroke” AND “upper limb impairments” AND “Intermittent theta burst stimulation” OR “theta burst stimulation”. Additional publications were discovered by looking through the reference lists of the retrieved articles. The search was conducted in November 2021.

### Inclusion and Exclusion Criteria

The inclusion criteria were: (1) participants had to be clinically diagnosed with upper limb dysfunction, and a CT scan or MRI was used to confirm a stroke; (2) iTBS, which contains a 2-s train of bursts of three 50-Hz pulses repeated every 200 ms (5 Hz) once a day during therapy, must be included in the intervention; (3) regular rehabilitative training was carried out without the use of iTBS or with the use of sham iTBS; and (4) the examination of upper limb function, which may include the evaluation of upper limb coordinated movement and separation movement, dexterity function and coordination function, must be included in outcome indicators. The primary outcome will be the Upper Limb Fugl-Meyer Assessment (FMA). Secondary outcomes will include brain impairment, participation, and quality of life measures; (5) randomized controlled trials (RCTs) or controlled trials should be used in all designs.

The following types of studies were excluded: (1) studies with non-primary data such as opinion articles, editorials, letters to the editor, and comments; (2) animal studies; (3) qualitative studies; (4) dissertations; and (5) studies that could not provide sufficient statistical data.

### Study Selection

**Figure 1:**
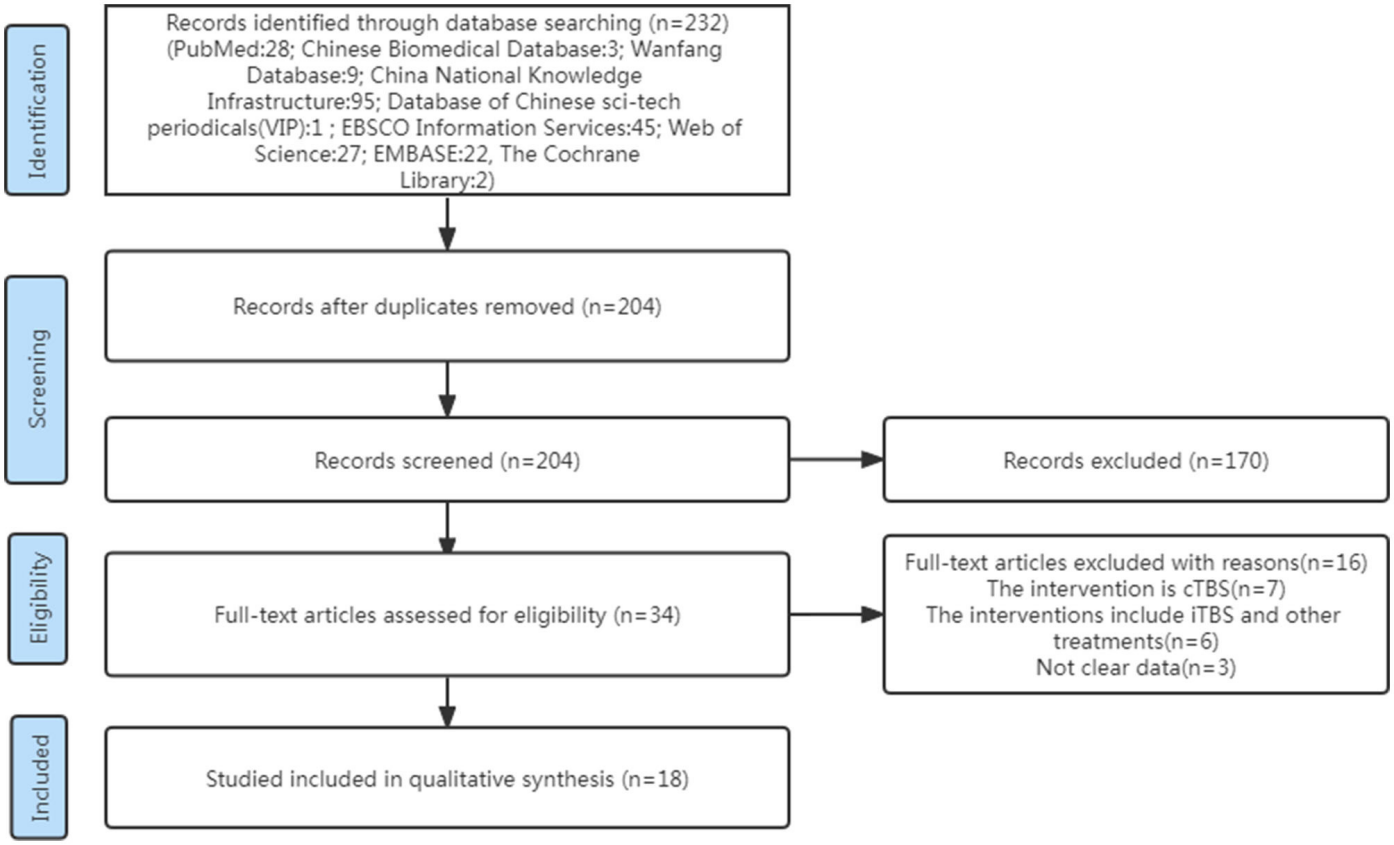
Flowchart for identification of studies.

Two independent researchers (HWH, Wen-Hao Huang and CJY, Jia-Yi Chen) carried out the screening method. Papers were screened for integrated analysis based on the inclusion and exclusion criteria given earlier to obtain eligible articles. Other relevant researches were found by screening reference lists manually. We compared the results of the two reviewers, and in the cases where the two reviewers could not agree, we sought the advice of the third reviewer (Ya-Dan Zheng) (see Figure 1).

### Data Extraction and Analysis

Data were extracted independently by the two reviewers (HWH and CJY) using a predesigned data extraction form, which included the following characteristics: articles (first author, publication year, and country), participants (sample size, age, gender, time since stroke, type of stroke, arm affected by stroke, and upper limb impairment), intervention (interventions treatment, comparisons treatment, stimulation parameters, and duration of treatment), and outcome measurement. If the data was not in the original journal, we looked for them on Clinical Trials (www.ClinicalTrials.gov) or contacted the authors. If there were several groups in the included RCTs, only those that were congruent with the systematic review's aims will be extracted. Team discussion was used to settle any discrepancies.

A meta-analysis will be conducted using Review Manager software (RevMan, version 5.3). The FMA score, MBI score, RMT, and latency of MEP were continuous measurement data, and the effect size was calculated using the mean difference (MD). After each research intervention, the MD was calculated using the mean, SD, and sample size; the incidence of adverse events was categorized as two-category count data, and the effect size was calculated using relative risk (RR). The number of incidents and sample size retrieved after each research intervention were used to calculate the RR. Calculate MD or RR and the corresponding 95% CI to estimate the total impact size of each indicator. The *p*-value and *I*^2^-value were quantified by finding the estimated value, and statistical heterogeneity was assessed using Cochrane Q statistics. Using the heterogeneity results, the appropriate effect model was selected. The fixed-effects model was used when *p* > 0.1 and *I*^2^ <50% and the random-effects model was used when *p* < 0.1 and *I*^2^ > 50%. If there was significant heterogeneity between studies, it was required to describe the source of the heterogeneity as much as possible, and subgroup analysis may be done based on the stimulus pattern and the different study subjects. To evaluate the stability and reproducibility of the results, perform a sensitivity analysis by eliminating each study separately. *p* ≤ 0.05 was statistically significant in the statistical analysis of all outcome markers.

The risk of bias was assessed independently by the two reviewers (HWH and CJY) using the Cochrane Collaboration risk of bias tool including the following items: random sequence generation, allocation concealment, blinding of participants and personnel, blinding of outcome assessment, incomplete outcome data, selective reporting, and other bias (14). According to the Cochrane Handbook for Systematic Reviews of Interventions, we categorized each included study's risk of bias as low, unclear, or high risk of bias. A third reviewer (ZYD) was involved if there were any disagreements.

## Result

### Study Characteristics

A total of 232 studies were identified in the initial search. A total of 204 articles were considered for screening. After duplicates were removed and abstracts screened, 34 studies remained for further assessment. After reviewing the full papers to obtain additional details and excluding articles for various reasons [the study's interventions were cTBS (*n* = 7); the study's interventions included iTBS and other treatments (*n* = 6); the study's data were not clear (*n* = 3)], 18 articles were included in the systematic review and meta-analysis. Many of the articles included in this study had an iTBS that delivered a 2-s train of bursts containing three 50-Hz pulses repeated every 200 ms (i.e., 5 Hz) at an intensity of 80% active motor threshold (AMT), (15–23) while the stimulus intensity of an article was 90% AMT (24). Furthermore, many articles cited a 70% resting motor threshold (RMT) (25–30) iTBS intervention intensity, although an article's stimulus intensity was 80% RMT (31) and another article's stimulus intensity was 100% RMT (32). Except for two publications that gave 1,200 pulses in total (17, 19), the majority of the articles in the study gave 600 pulses total for iTBS intervention (see Table 1).

**Table 1:** Summary of the included studies and the detail of intervention and measurement.

**Reference**	**Subjects**		**Age (years)**		**Type of interventions**		**Time of interventions**	**Outcomes measures**
	**T**	**C**	**T**	**C**	**T**	**C**		
Chen et al. (15)	11	11	52.9 ± 11.1	52.6 ± 8.3	iTBS + routine rehabilitation	Sham - iTBS + routine rehabilitation	5d/wk, 2wk	MAS, FMA-UE, ARAT, BBT, MAL
Ackerley et al. (24)	9	9	(21–80)	(38–79)	iTBS + routine rehabilitation	Sham - iTBS + routine rehabilitation	5d/wk, 2wk	FMA-UE, ARAT
Liao et al. (32)	22	21	55.86 ± 9.12	59.52 ± 13.11	iTBS + routine rehabilitation + medical therapy	Routine rehabilitation + medical therapy	6d/wk, 2wk	FMA-UE, MAS, MBI
Sung et al. (16)	12	14	64.2 ± 11.9	63.1 ± 12.8	iTBS + routine rehabilitation	Sham - iTBS + routine rehabilitation	5d/wk, 4wk	FMA-UE, WMFT, RMT, MEP amplitude, MEP latency
Watanabe et al. (31)	8	6	72.5 ± 6.5	75.2 ± 5.5	iTBS + routine rehabilitation	Sham - iTBS + routine rehabilitation	10 consecutive days	FMA-UE, MAS, MEP amplitude
Volz et al. (25)	13	13	69.69 ± 12.99	64.69 ± 13.26	iTBS intervention over the ipsilesional primary motor cortex (M1) + routine rehabilitation	iTBS intervention over the parieto-occipital vertex + routine rehabilitation	5 consecutive days	The relative grip strength, JTT
Hsu et al. (17)	6	6	56.8 ± 6.8	62.3 ± 8.5	iTBS + routine rehabilitation + medical therapy	Sham - iTBS + routine rehabilitation + medical therapy	10 consecutive days	UE-FMT, ARAT, NIHSS, AMT, RMT, MEP amplitude
Tang et al. (26)	8	8	53.75 ± 10.77	55.62 ± 14.55	iTBS + routine rehabilitation	Sham - iTBS + routine rehabilitation	5d/wk, 2wk	FMA-UE, MSS, BI, RMT
Yu et al. (27)	15	14	51.60 ± 12.78	55.57 ± 9.43	iTBS + routine rehabilitation	Routine rehabilitation	5d/wk, 2wk	FMA-UE, MAS, MBI, MMSE, RMT
Jiang et al. (28)	13	13	61.31 ± 11.25	51.843 ± 11.58	iTBS + routine rehabilitation + medical therapy	Routine rehabilitation + medical therapy	10 consecutive days	FMA-UE, ARAT, MBI, MEP amplitude, MEP latency
Chen et al. (18)	16	16	57.38 ± 8.04	51.44 ± 9.19	iTBS + routine rehabilitation	Sham - iTBS + routine rehabilitation	5d/wk, 2wk	MAS, MTS, SWV, MEP amplitude, MEP latency
Chen(2) et al. (19)	12	11	54.36 ± 10.56	48.95 ± 9.63	iTBS + VCT	Sham - iTBS + VCT	5d/wk, 3wk	MAS, FMA-UE, ARAT, BBT
Zhang et al. (29)	12	12	-	-	iTBS + RAT + routine rehabilitation	Sham - iTBS + RAT + routine rehabilitation	3-5d/wk, 2-3wk	FMA-UE, ARAT, EEG
Ding et al. (30)	15	15	65.1 ± 11.9	61.1 ± 12.1	iTBS	Sham - iTBS	-	FMA-UE, ARAT, EEG
Kim et al. (20)	15		60.7 ± 8.7		iTBS and sham- iTBS were separated by a 1-week		-	MAS, MTS
Talelli et al. (21)	13	12	54.4 ± 15.8	58.5 ± 12.0	iTBS + routine rehabilitation	Sham - iTBS + routine rehabilitation	5d/wk, 2wk	ARAT, BI, JTT, 9HPT, VAS
Li et al. (22)	4	4	57.28 ± 14.69	55.72 ± 14.12	iTBS + electroacupuncture	Sham - iTBS + electroacupuncture	5 consecutive days	FMA-UE, RMT
Zhou et al. (23)	6	6	62.67 ± 8.52	47.33 ± 17.94	iTBS + routine rehabilitation	Routine rehabilitation	5d/wk, 2wk	FMA-UE, NIHSS, MBI, RMT, MEP amplitude, MEP latency

*FMA–UE, Fugl-Meyer Assessment-Upper Extremity; BI, Barthel Index; MBI, Modified Barthel Index; NIHSS, the National Institutes of Health Stroke Scale; WMFT, Wolf motor function test; BBT, Box and Block test; RMT, Resting exercise threshold; AMT, Active exercise threshold; MAS, modified Ashworth scale; ARAT, the action research arm test; MAL, the motor activity log; MSS, motor status scale; NHPT, the nine hole peg test; JTT, Jebsen–Taylor Hand Function Test; MTS, Modified Tardieu Scale; SWV, Shear Wave Ultrasound Elastography; VCT, virtual reality-based cycling training; RAT, Robot-assisted training; EEG, electroencephalography; VAS, visual analog scale*.

### Baseline of Patients

In total, 401 stroke patients of mixed gender were included in the study. According to the included articles, the participants ranged in age from 18 to 90 years old, and the average age of the patients included in the study was 52.22 ± 9.91 (mean = 52.22, SD = 9.91). The gender distribution of the studies was 109 women and 268 men. The onset time of all subjects included in the study may be as short as 2 weeks or as long as 6 months or more. The average onset time of the patients included in the study is 5.73 ± 4.95 months (mean = 5.73, SD = 4.95) (see Table 2).

**Table 2:** Characteristics of studies included in the meta-analysis.

**Studies**	**Study design**	**Stroke duration (Mean ±SD)**	**Gender (Male/Female)**	**Side of effect** **(Left/Right)**	**Intensity, frequency and pulse length**	**Stimulation location**	**Direction of effects (+ positive, - negative, +/- both)**
Chen et al. (15)	Clinical Trial	≥6 months	14/8	15/7	80%AMT, 50Hz/5Hz, 600 pluses	M1	+/-
Liao et al. (32)	Clinical Trial	10 days- 1 year	35/8	26/17	100%RMT, 50Hz/5Hz, 600 pluses	M1	+/-
Sung et al. (16)	Randomized Controlled Trial	3 months- 12 months, 8.2 ± 1.6 months	20/6	-	80%AMT, 50Hz/5Hz, 600 pluses	M1	+/-
Hsu et al. (17)	Randomized Controlled Trial	2 weeks- 4 weeks, 21.4 ± 4.5 days	8/4	8/4	80%AMT, 50Hz/5Hz, pluses	M1	+/-
Tang et al. (26)	Clinical Trial	1month-6months, 52.25 ± 24.03 days	14/2	14/2	70%RMT, 50Hz/5Hz, 600 pluses	M1	+/-
Yu et al. (27)	Clinical Trial	15 days- 6 months, 77.93 ± 45.15 days	24/5	12/17	70%RMT, 50Hz/5Hz, 600 pluses	M1	+/-
Chen et al. (18)	Randomized Controlled Trial	2 weeks- 6 months, 90.82 ± 44.67 days	25/7	19/13	80%AMT, 50Hz/5Hz, 600 pluses	Cerebellar	+/-
Ding et al. (30)	Clinical Trial	≤ 18months, 3.95 ± 3.7 months	21/9	18/12	70%RMT, 50Hz/5Hz, 600 pluses	M1	+/-
Talelli et al. (21)	Clinical Trial	≥1 year, 27.58 ± 30.11 months	16/9	10/15	80%AMT,50Hz/5Hz, 600 pluses	M1	+/-
Zhou et al. (23)	Clinical Trial	2 weeks-1 month, 27.09 ± 3.34 days	11/1	4/8	80%AMT,50Hz/5Hz, 600 pluses	M1	+

###  Risk of Bias of Included Studies

**Figure 2:**
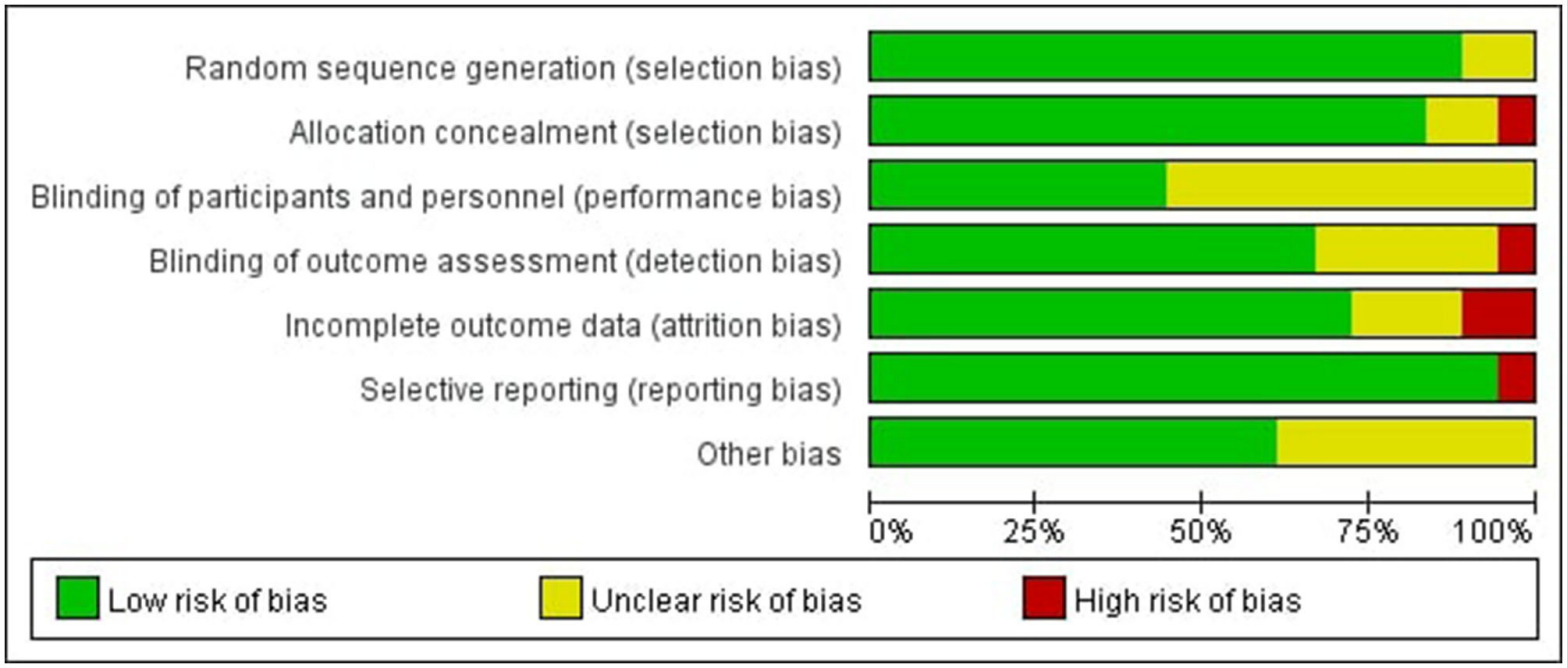
Risk of bias graph.

**Figure 3:**
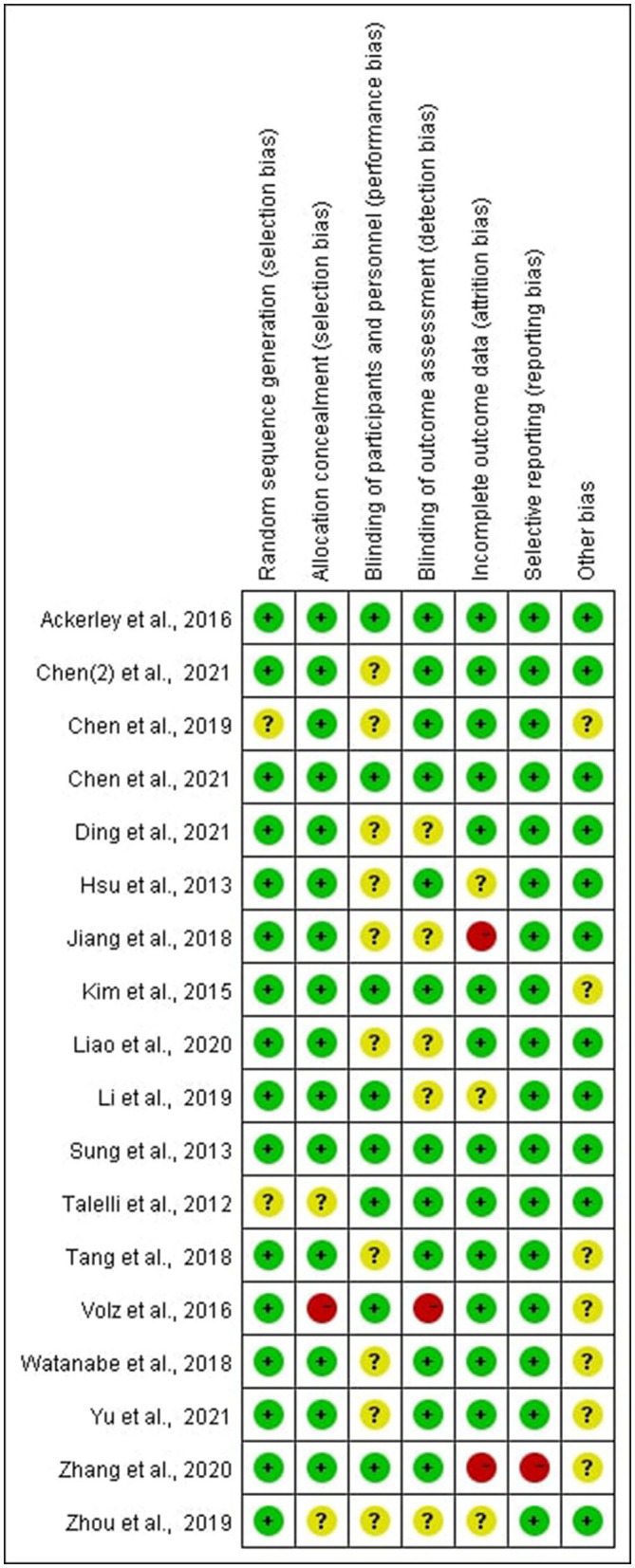
Risk of bias summary.

In all of the trials considered, the items' selective reporting were rated as low risk of bias except one (29). Random sequence generation or allocation concealment scored a high risk of bias or unclear risk in four of the included studies, which led to the error of the result to increase (15, 21, 23, 25). Blinding of participants and personnel scored unclear risk in some of the included studies, which was inherent to the intervention (15, 17, 18, 23, 26–28, 30–32). According to certain studies, the blinding of outcome assessment was not specified adequately, resulting in a score that was unclear or high in the detection bias (22, 23, 25, 28, 30, 32). In some studies, incomplete outcome data were scored as high risk of bias or unclear risk, indicating that the data was incomplete or missing (17, 22, 23, 28, 29) (see Figures 2, 3).

### Meta-Analysis Outcomes

To assess the effect of iTBS on upper limb function, post-intervention data from ten trials involving 247 participants were pooled. The results of this analysis indicated that iTBS had a significant effect on the motor function of stroke patients' upper limbs. In addition, there was a significant positive correlation between iTBS intervention and activities of daily living. And the results of this meta-analysis also indicated that the assessment of RMT and MEP latency time was significantly different in the iTBS group.

#### Motor Function

**Figure 4:**
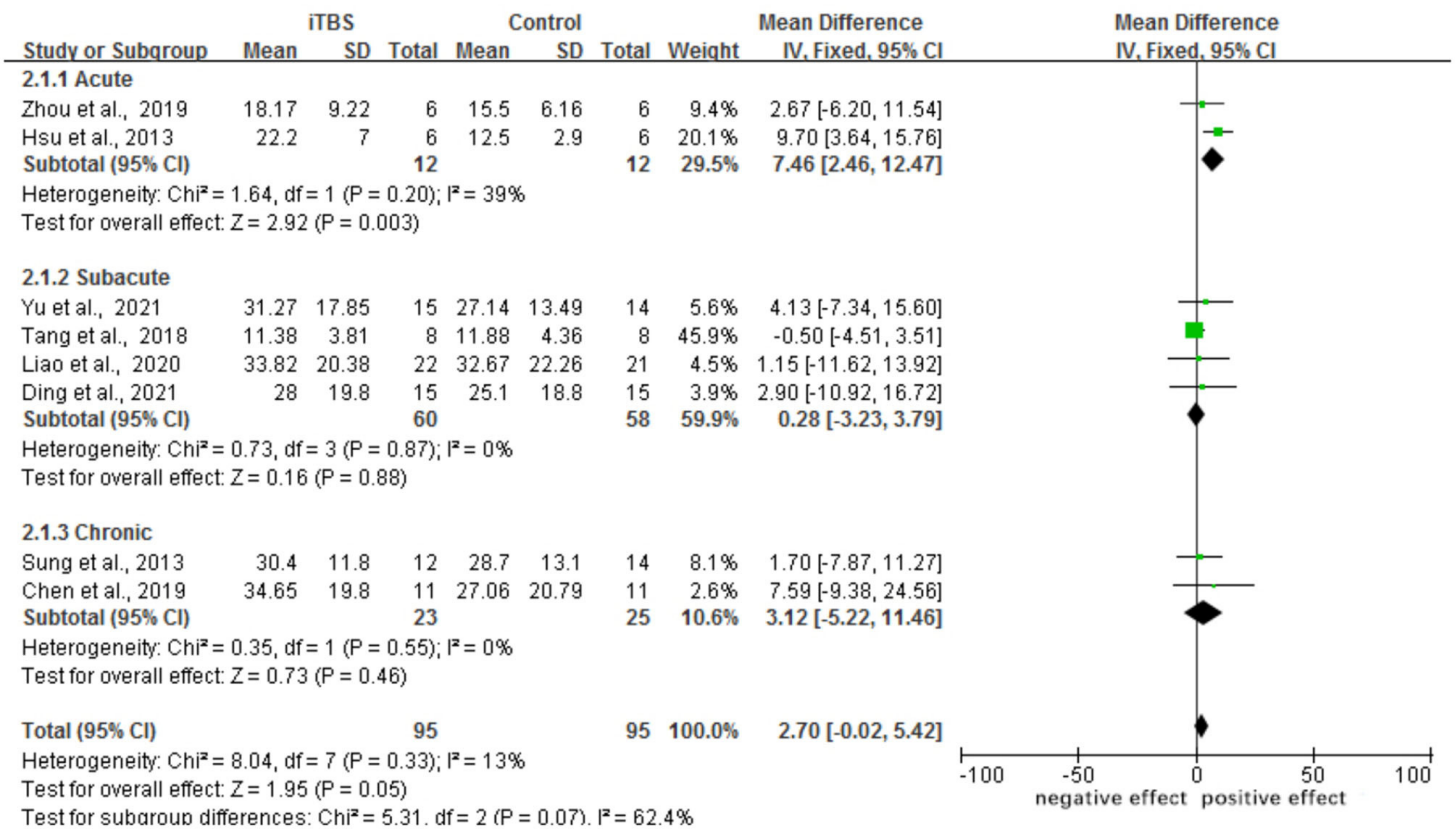
Forest plots of the pooled results on motor function.

**Figure 5:**
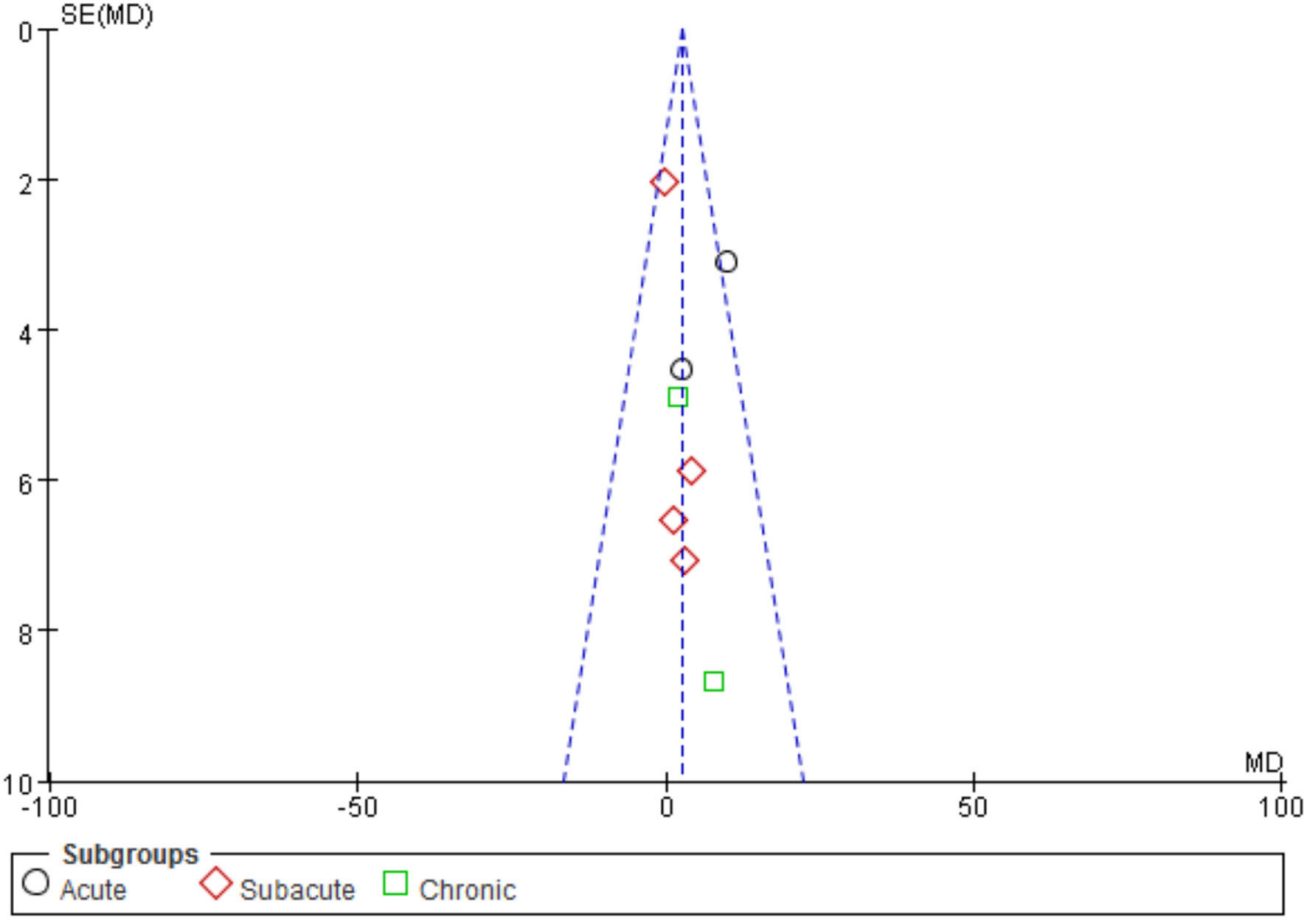
Funnel plots of the pooled results on motor function.

A total of eight trials involving 192 individuals were chosen. The meta-analysis revealed a significant difference in FMA scores between the iTBS and control groups (MD 2.70, 95% CI −0.02 to 5.42, *p* = 0.05). The mean effect size for the acute subgroup was 7.46 (95% CI: 2.46 to 12.47; *p* = 0.003) and homogeneous (*I*^2^ = 39.0%). The mean effect size for the subacute subgroup was 0.28 (95%CI: −3.23–3.79; *p* = 0.88), with no evidence of heterogeneity (*I*^2^ = 0.0%). The mean effect size for the chronic subgroup was 3.12 (95% CI, −5.22 to 11.46; *p* = 0.46), with was no evidence of significant heterogeneity (*I*^2^ = 0.0%). From the results of iTBS intervention in patients with various post-stroke complications, we conjecture that iTBS is most effective in improving motor function in the acute phase after the intervention. Refer to Figure 4. A funnel plot was used to analyze publication bias. According to reports, if a study's data falls outside of the 95% range, it is deleted from the funnel plot. The funnel plot appears to be symmetrical, with all the studies falling within the 95% CI, indicating that there is no publication bias see Figure 5.

#### Activities of Daily Living

**Figure 6:**
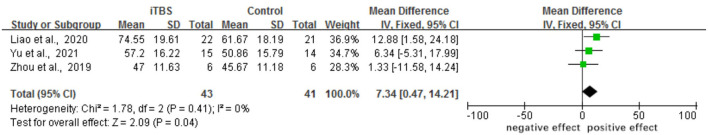
Forest plots of the pooled results on daily living.

The results of this meta-analysis indicated that when the iTBS group was compared with the control group, the assessment of MBI was significantly different (MD 7.34, 95% CI 0.47–14.21, *p* = 0.04). One trial found that the iTBS group did not significantly improve their BI score when compared with the control group (23). Although a five-point rating system replaced the original two, three, or four-point rating system in BI in the MBI, the article's findings are still worth referencing (see Figure 6).

#### Electrophysiological Measures

**Figure 7:**
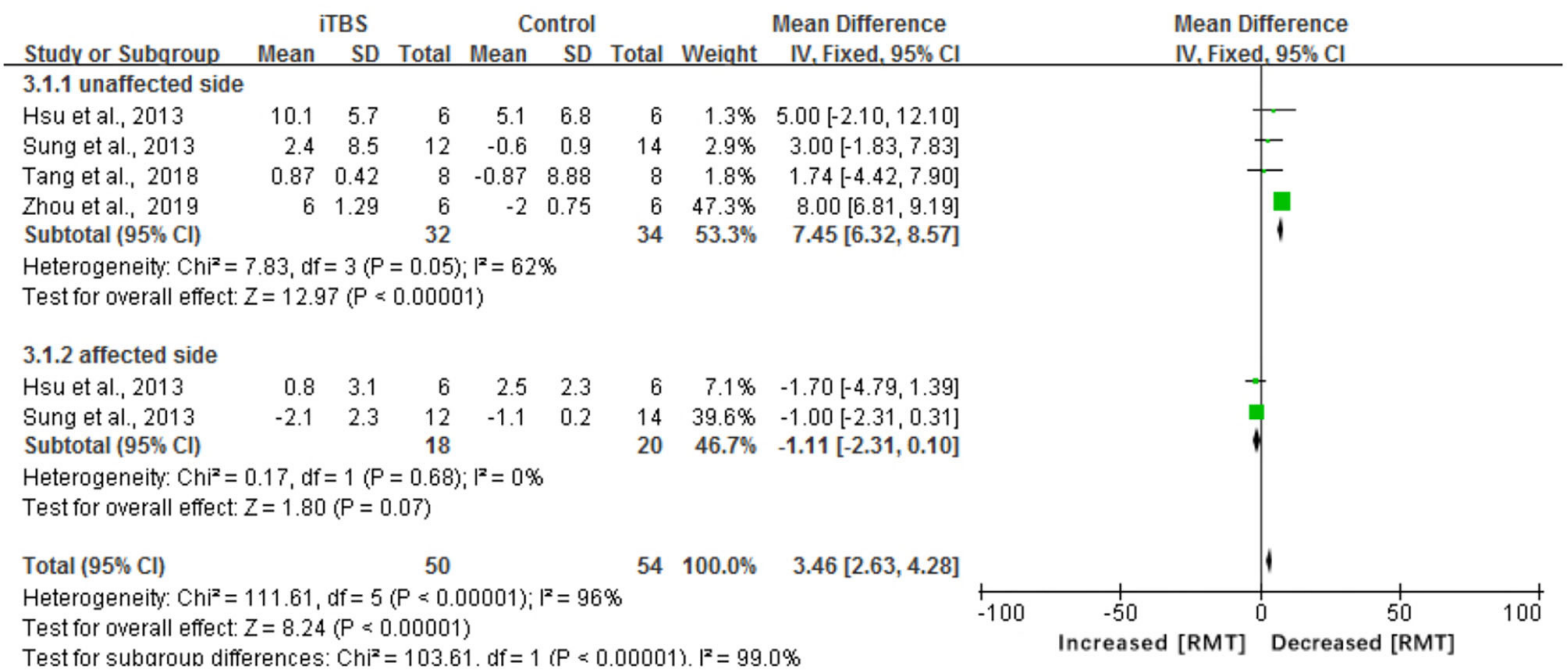
Forest plots of the pooled results on RMT.

**Figure 8:**
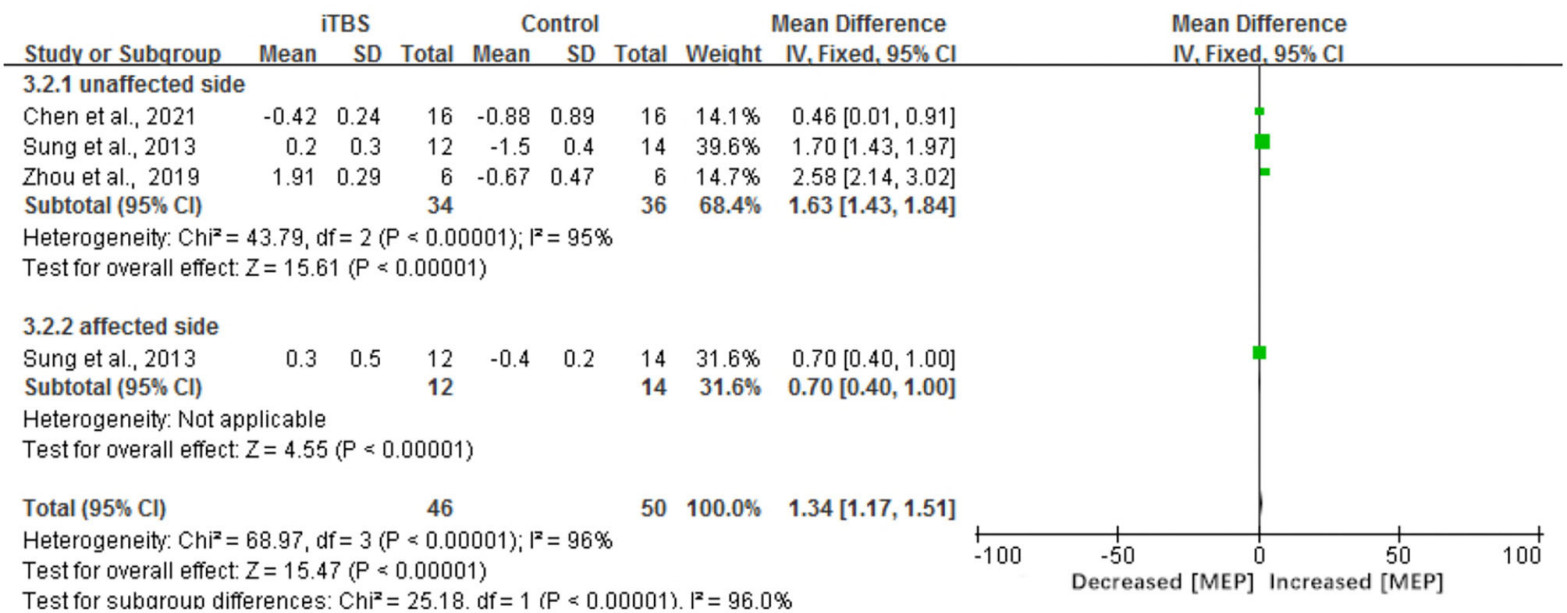
Forest plots of the pooled results on the latency of MEP.

The results of our meta-analysis showed that when compared with the control group, the assessment of RMT had a significant difference in the iTBS group (MD 3.46, 95% CI 2.63–4.28, *p* < 0.00001) (see Figure 7). Besides, there was a significant improvement in the time of latency of MEP(MD 1.34, 95% CI 1.17 to 1.51, *p* < 0.00001) (see Figure 8). There was a trend of increased corticomotor excitability on the unaffected side and decreased corticomotor excitability on the affected side after iTBS interventions.

## Discussion

iTBS has emerged as a novel method for improving upper extremity function in stroke patients. We conducted a systematic review and meta-analysis to determine the efficacy of iTBS training on limb function in stroke patients.

The study included a total of 18 publications and 401 subjects. After analyzing the included articles, we discovered that therapy with iTBS resulted in better treatment outcomes than the control group. The majority of publications subjected to meta-analysis revealed a range of outcomes, both positive and negative. Only one study reported an entirely positive conclusion (23). FMA–UE, MBI, RMT, MEP amplitude, and MEP latency were used as outcome tests in this study for stroke patients during the acute phase. According to our findings, post-stroke patients' FMA scores improved after iTBS intervention and were most pronounced in the acute phase. However, the change in FMA score was merely 2.7 points, which is relatively minimal for the improvement of the patient's upper limb function. Besides, since the sample size we used was limited, reaching this result is not very persuasive. More research is required in the future to corroborate this conclusion. According to the included studies, it was widely believed that iTBS delivered a 2-s train of bursts consisting of three 50-Hz pulses repeated every 200 ms (5 Hz) at an intensity of 70–80% AMT or RMT every 10 s for 20 times (600 pulses total), which was the most beneficial plan (15, 16, 18, 21, 23, 26, 27, 30). At the same time, the majority of studies had positive benefits after 2 weeks or 10 sessions, which warrants further investigation (15, 17–19, 21–28, 31, 32).

According to the included studies, patients' FMA scores slightly improved and MBI scores improved significantly following iTBS intervention treatment, as did their ability to participate in activities. Diekhoff Krebs' study demonstrated that iTBS stimulation of ipsilesional M1 resulted in a significant increase in MEP in the stimulated hemisphere and a significant decrease in the unstimulated hemisphere (33). John Rothwell and Hamada proved that, based on electrophysiological biomarkers of latency, the effects of TBS protocols can occasionally differ from those originally reported and that individual's responses can be considerably a variable (34). It has been established that rTMS could enable post-stroke patients to improve their self-care abilities. Disinhibition of the uninjured cerebral hemisphere following a stroke can result in daily function limitations and has been associated with a lower rate of functional recovery. On the other hand, iTBS as a form of rTMS, used a similar mechanism to balance this interhemispheric interaction, thereby improving the patient's daily self-care abilities (35). As a result, our meta-analysis could account for the slight improvement in FMA scores and the significant improvement in MBI scores in the stroke patients following iTBS treatment. In addition, when different sites are stimulated, it can improve motor function by activating different pathways. The use of iTBS to stimulate the cerebellum has been shown to improve visual–motor integration, which has been shown to improve upper limb function in the stroke patients (18).

There were some limitations in this study. First, the articles we evaluated for analysis had a variety of patient demographic features, such as the age, gender, stroke duration, and stroke side of the stroke patient. When patients had varying stroke duration, there was significant heterogeneity in motor recovery after stimulation (36). From our analysis of the FMA scores of patients after a stroke at different onset times, it can be concluded that patients in the acute phase after iTBS had a more significant improvement in motor function compared with the subacute phase and the chronic phase. However, in the articles we included, most authors did not specify the type of stroke, the patients of three studies with ischemic stroke (17, 25, 32) and one with hemorrhagic stroke (26), and the others included patients with ischemic or hemorrhagic stroke, so we could not judge whether different stroke location had an impact on the results. Second, the sample size of the patients included in the experiment was insufficient, resulting in a bias in the results reported in different journals (37). It reminded us that we would need to collect additional patients for a larger sample size investigation. Third, the majority of the iTBS treatments included in this analysis were set at 600 pulses, but one of the studies was set at 1,200 pulses (17). For the study of 1,200 pulses, although the FMA score after iTBS treatment showed a clear significance, the data from this article alone cannot support this conclusion. So, we need to do more research on the contrast between different pulses in the future. As a result of the variable number of pulses, patients may have varying degrees of functional improvement after treatment. Finally, the majority of the studies included in the review did not fully explain information on randomization, concealment, blinding, and other factors, which might have influenced the analysis' reliability.

## Conclusion

This review has provided supportive evidence for the efficacy of iTBS in improving post-stroke patients' brain excitability, upper limb function, and daily life. Additional research with a larger sample size would be necessary to fully characterize the role of iTBS as a neurorehabilitation treatment for upper limb dysfunction. Future research is necessary to compare various stimulation parameters, such as stimulation site, frequency, and duration, to determine the optimal treatment protocol.

## Data Availability Statement

The original contributions presented in the study are included in the article/supplementary material, further inquiries can be directed to the corresponding author.

## Author Contributions

WH and JC conceived and designed the study and wrote the manuscript. WH, JC, and ZD developed the search strategy. WH, ZD, and JZ screened abstracts and full text reports. JC, XL, and LS extracted outcomes. YL and ZD interpretation of the data. All authors contributed to the article and approved the submitted version.

## Funding

This work was supported by the National Natural Science Foundation of China (Grant No. 81972159), the Natural Science Foundation of Guangdong Province, China (Grant No. 2020A1515010881), and Shenzhen Science technology project (Grant No. JSGG20201102145602006).

## Conflict of Interest

The authors declare that the research was conducted in the absence of any commercial or financial relationships that could be construed as a potential conflict of interest.

## Publisher's Note

All claims expressed in this article are solely those of the authors and do not necessarily represent those of their affiliated organizations, or those of the publisher, the editors and the reviewers. Any product that may be evaluated in this article, or claim that may be made by its manufacturer, is not guaranteed or endorsed by the publisher.
